# Efficacy Comparison of LPA_2_ Antagonist H2L5186303 and Agonist GRI977143 on Ovalbumin-Induced Allergic Asthma in BALB/c Mice

**DOI:** 10.3390/ijms23179745

**Published:** 2022-08-28

**Authors:** Ye-Ji Lee, Dong-Soon Im

**Affiliations:** Department of Basic Pharmaceutical Sciences, Graduate School, Kyung Hee University, Seoul 02446, Korea

**Keywords:** asthma, allergy, H2L5186303, GRI977143, LPA_2_, lysophosphatidic acid

## Abstract

Lysophosphatidic acid (LPA), an intercellular lipid mediator, is increased in the bronchoalveolar fluids of patients with asthma after allergen exposure. LPA administration exaggerates allergic responses, and the type 2 LPA receptor (LPA_2_) has been reported as a therapeutic target for asthma. However, results with LPA_2_ agonist and antagonist along with LPA_2_ gene deficient mice have been controversial and contradictory. We compared the effects of LPA_2_ antagonist (H2L5186303) and agonist (GRI977143) in a single experimental protocol of ovalbumin (OVA)-induced allergic asthma by treating drugs before antigen sensitization or challenge. H2L5186303 showed strong suppressive efficacy when administered before OVA sensitization and challenge, such as suppression of airway hyper responsiveness, inflammatory cytokine levels, mucin production, and eosinophil numbers. However, GRI977143 showed significant suppression when administered before an OVA challenge. Increases in eosinophil and lymphocyte counts in the bronchoalveolar lavage fluid, Th2 cytokine levels, inflammatory scores, and mucin production were differentially ameliorated by the two drugs. The results demonstrate the multiple roles of LPA_2_ in asthmatic responses. We suggest that the development of LPA_2_ antagonists would achieve better therapeutic efficacy against asthma than agonists.

## 1. Introduction

Asthma is a chronic inflammatory condition characterized by swollen and narrow airways [1]. Affected patients generally experience shortness of breath, chest tightness, coughing, and wheezing [1]. Pathological observations include eosinophil infiltration, mucus hyperproduction, and bronchial mucosal thickening and wall remodeling [1]. Asthmatic symptoms are usually controlled by corticosteroids, leukotriene D_4_ antagonists, and long-acting β_2_ adrenoceptor agonists [2]. However, drug-resistant asthma and steroid-induced side effects require novel approaches to overcome the limitations of such therapies [2].

Lysophosphatidic acid (LPA) is a simple lipid mediator with variable acyl chain lengths. Studies suggest that LPA levels (22:5 and 22:6) are significantly increased in the bronchoalveolar lavage fluid (BALF) of asthmatic patients and mice challenged with segmental allergen and house dust mite (HDM), respectively [3,4,5]. Additionally, LPA inhalation induces histamine release and enhances eosinophil and neutrophil infiltration into the lung alveolar spaces in guinea pigs [6,7]. Pro-inflammatory functions of LPA in immune cells have also been reported. According to a study, LPA induces calcium mobilization, actin reorganization, and chemotaxis in human eosinophils [8]. It also induces histamine release [9] and chemokine generation via IL-4 in human mast cells [10]. Moreover, it also enhances IL-13 gene expression in Th2 cells in vitro [11,12]. In contrast, LPA attenuates cytokine secretion in human and murine dendritic cells [13,14]. LPA stimulates the expression of thymic stromal lymphopoietin and CCL20 [15], and IL-8 production in primary cultured human bronchial epithelial cells by activating p38 MAP kinase and JNK [15,16]. In addition, the expression of LPA_2_ was higher than LPA_1_ in naïve T cells [5], and in mouse tracheal epithelial cells LPA_2_ mRNA showed the highest expression among LPA_1–4_ [17]. Moreover, LPA_2_ deficiency protects against bleomycin-induced lung injury and fibrosis [18].

The involvement of LPA in allergic asthma and LPA_2_ expression in epithelial and immune cells led to the investigation of the functional roles of LPA_2_ in allergic asthma. Zhao et al. used a murine model of *Schistosoma mansoni* egg sensitization and challenged heterozygous LPA_2_ gene deficient mice (LPA_2_^+/−^). Results demonstrated a reduced influx of eosinophils and periodic acid-Schiff (PAS)-positive cells in LPA_2_^+/−^ compared to wild-type mice, suggesting a pro-inflammatory role of LPA_2_ in allergic asthma development [17]. However, Emo et al. reported that OVA-sensitized and challenged homozygous LPA_2_ gene-deficient mice (LPA_2_^−/−^) showed more severe lung inflammation than wild-type mice, implying a negative regulation by LPA_2_ signaling [19]. In contrast, attenuation of allergic lung inflammation and Th2 cytokines was observed in LPA_2_^−/−^ mice challenged with three allergen (HDM, ragweed, and *Aspergillus* sp.) [4]. Previous studies suggest that LPA_2_ antagonist H2L5186303 ((Z,Z)-4,4′-[1,3-phenylenebis(oxy-4,1-phenyleneimino)] bis [4-oxo-2-butenoic acid]) suppress allergic airway inflammation [5,20]. However, the LPA_2_ agonist DBIBB (2-[4-(1,3-dioxo-1H,3H-benzoisoquinolin-2-yl)butylsulfamoyl] benzoic acid) also suppresses allergic eosinophilic bronchial inflammation [21]. Nevertheless, differences in asthma-inducing antigens, experimental protocols, and mouse genetic strains may lead to contradictory and controversial results. Therefore, we compared the effects of an LPA_2_ antagonist (H2L5186303) and an LPA_2_ agonist (GRI977143) on OVA-induced allergic asthma by providing drug treatment before antigen sensitization or challenge.

## 2. Results

### 2.1. GRI977143 and H2L5186303 Suppressed Degranulation of Mast Cells

**Figure 1:**
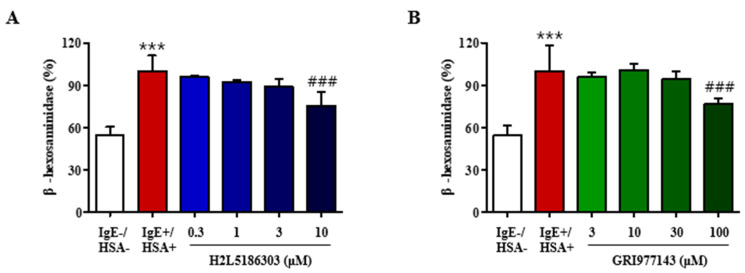
H2L5186303 inhibits antigen-induced degranulation in RBL-2H3 mast cells. After sensitization with anti- dinitrophenyl immunoglobulin E (DNP-IgE) for 18 h, RBL-2H3 cells were challenged with dinitrophenyl-human serum albumin (DNP-HSA). H2L5186303 (**A**) and GRI977143 (**B**) treatment was performed at the indicated concentrations 30 min before antigen challenge. Basal degranulation shows samples without IgE and HSA, and the positive control shows samples with IgE and HSA. The results are presented as the mean ± standard error (SE) of three independent experiments. *** *p* < 0.001 vs. the HSA-untreated group. ### *p* < 0.001 vs. the HSA-treated group.

**Figure 2:**
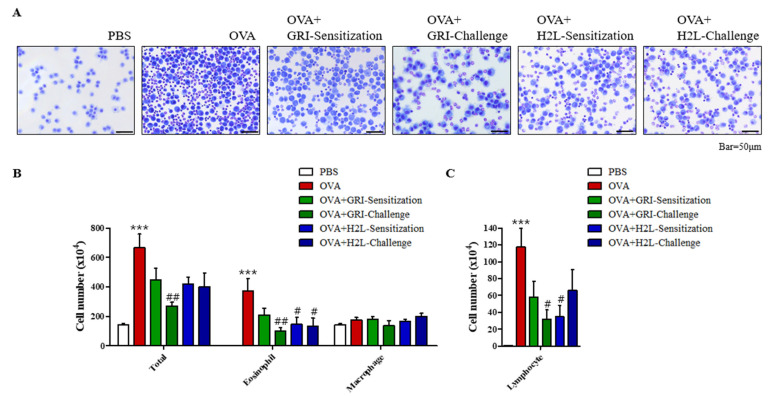
H2L5186303 and GRI977143 inhibit ovalbumin-induced immune cell accumulation in BALF. (**A**) Mice were sensitized with ovalbumin (OVA) twice via intraperitoneal injection on days 0 (D0) and 14 (D14) and challenged on D28, D29, and D30 with nebulized OVA. H2L5186303 or GRI977143 was administered intraperitoneally at a dose of 1 mg/kg 30 min before OVA sensitization or challenge. BALF cells were stained with May–Grünwald stain and counted. (**B**) Total cell, macrophage, and eosinophil counts in BALF. (**C**) Lymphocyte counts in BALF. The results are presented as mean ± standard error (SE) of the cell count values (*n* = 5). *** *p* < 0.001 vs. the phosphate buffered saline-treated group, # *p* < 0.05, ## *p* < 0.01 vs. the OVA-treated group.

Antigen-induced cross-linking of the IgE-FcεRI complex activates mast cells and induces mast cell degranulation, which is the initial step in eliciting asthmatic response via release of histamine, leukotrienes, and neutral proteases. We measured the degranulation response in RBL-2H3 rat basophilic leukemia cells by assessing β-hexosaminidase activity after exposure to human serum albumin (antigen) (Figure 1A). Results revealed that H2L5186303 treatment inhibited β-hexosaminidase release in a concentration-dependent manner (Figure 1A), implying that LPA functions as a mast cell activator via LPA_2_. On the contrary, GRI977143 also inhibited inhibition of β-hexosaminidase release at 100 µM concentration, which was ten-fold higher than the effective concentration of H2L5186303 (10 µM) (Figure 2A). This may imply a non-specific off-target effect of the LPA_2_ agonist, GRI977143 (Figure 2A).

### 2.2. GRI977143 and H2L5186303 Suppressed the Elivated Eosinophil and Lymphocyte Levels

In an OVA-induced mouse model of asthma, GRI977143 and H2L5186303 were intraperitoneally injected 30 min before OVA sensitization or challenge. As a result, an elevated total cell count in BALF was suppressed (Figure 2A). The total cell count in BALF increased to 368.5% in the OVA-induced asthma compared to the PBS-treated control group (Figure 2B); however, GRI977143 treatment before antigen challenge significantly suppressed the OVA-induced increase in total cell count by 75.5% (Figure 2B). In addition, the immune cell population distribution was also assessed. Specifically, GRI977143 treatment before challenge significantly decreased the OVA-induced increase in eosinophil counts by 72.5% (Figure 2B). Furthermore, H2L5186303 treatment before antigen sensitization and challenge significantly suppressed the OVA-induced increase in eosinophil counts by 60.9% and 63.7%, respectively. Moreover, an elevated lymphocyte count in response to OVA treatment was significantly decreased by 73.4% and 70.7% by GRI977143 treatment before challenge and H2L5186303 treatment before sensitization, respectively (Figure 2C). However, macrophage count was not significantly increased by OVA treatment.

### 2.3. GRI977143 and H2L5186303 Suppressed AHR

**Figure 3:**
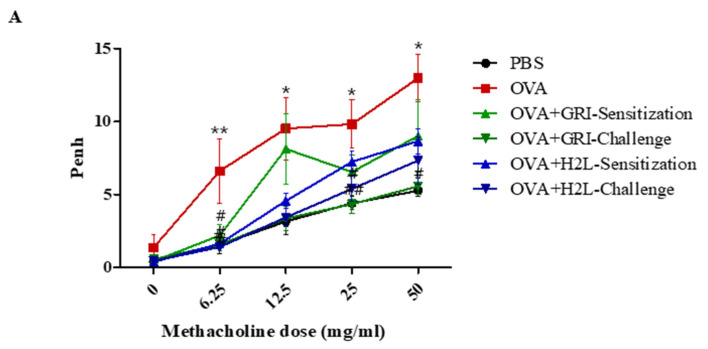
H2L5186303 and GRI977143 suppressed OVA-induced airway hyper responsiveness. The effects of H2L5186303 and GRI977143 on AHR were measured using noninvasive whole-body plethysmography. Values are presented as the mean ± SEM of three independent experiments. # *p*  <  0.05, ## *p*  <  0.01 vs. control group; * *p*  <  0.05, ** *p*  <  0.01 vs. OVA group.

The effects of GRI977143 and H2L5186303 on AHR were measured. Results showed that the Penh value of the OVA-challenged mice was higher than the control group. A significantly reduced AHR was observed in OVA-challenged mice treated with GRI977143 before OVA challenge but not sensitization (Figure 3). Moreover, H2L5186303 treatment before OVA sensitization and challenge significantly reduced AHR in OVA-challenged mice (Figure 3).

### 2.4. GRI977143 and H2L5186303 Suppressed Mucin Secretion and Lung Inflammation

**Figure 4:**
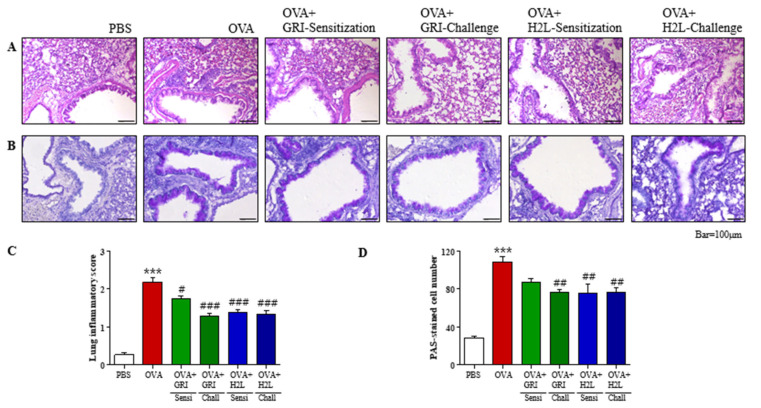
H2L5186303 and GRI977143 protect against airway inflammation and mucin production. (**A**) Panels show H&E-stained sections of lung tissues from the phosphate-buffered saline (PBS), ovalbumin (OVA), H2L5186303-treated OVA (before sensitization or challenge), and GRI977143-treated OVA groups (before sensitization or challenge). Small dark blue dots around the bronchioles indicate eosinophils, which were scarce in the PBS but densely accumulated around the bronchioles in the OVA group. However, eosinophil accumulation was less obvious in the OVA + H2L5186303 and GRI977143 groups than in the OVA group. (**B**) Panels show PAS/hematoxylin-stained sections of lung tissues from the PBS, OVA, H2L5186303-treated OVA (before sensitization or challenge), and GRI977143-treated OVA groups (before sensitization or challenge). PAS staining showed that mucin was stained purple. A darker and denser purple color surrounding the bronchioles was observed in the OVA group but not in the PBS group. (**C**) Lung inflammation was semi-quantitatively evaluated, and histological findings were scored as described in the Materials and Methods section. (**D**) Mucous production was measured by counting the number of PAS-positive cells per millimeter of bronchiole (*n* = 5 per group). Values represent mean ± standard error (SE) (*n* = 5). *** *p* < 0.001 vs. the PBS-treated group, # *p* < 0.05, ## *p* < 0.01, ### *p* < 0.001 vs. the OVA-treated group.

Histological studies using H&E and PAS staining of lung samples showed an increased inflammation and mucin secretion. Eosinophils were observed as small, dark blue dots in H&E staining of the lung sections (Figure 4A). There were few eosinophils in the PBS control compared with those observed around the bronchioles in the OVA group (Figure 4A). GRI977143 before OVA challenge and H2L5186303 before OVA sensitization or challenge inhibited OVA-induced elevated eosinophil counts (Figure 4A). Semi-quantitative evaluation of lung inflammation (using a scale of 0–3) indicated an average inflammation score of 2.168 in the OVA-treated group, which was significantly reduced after GRI977143 and H2L5186303 treatments before the challenge (Figure 4C).

Mucin and mucous glycoproteins produced by goblet cells were visualized by PAS staining of the lung sections. Mucin-secreting cells were observed as dark violet spots surrounding bronchioles in the OVA group (Figure 4B). PAS staining was inhibited after GRI977143 treatment before the OVA challenge and H2L5186303 treatment before OVA sensitization or challenge (Figure 4B). Furthermore, mucin production was estimated by counting the number of PAS-positive cells in the bronchioles (Figure 4D). Results demonstrated that stained cells were scarce in the PBS-treated group; however, approximately 100 PAS-positive cells/mm were detected in the OVA-treated group, which were significantly reduced after GRI977143 and H2L5186303 treatments before the OVA challenge (Figure 4D).

### 2.5. GRI977143 and H2L5186303 Suppressed the Production of Th2 and Th1 Cytokines in the BALF and Lungs

**Figure 5:**
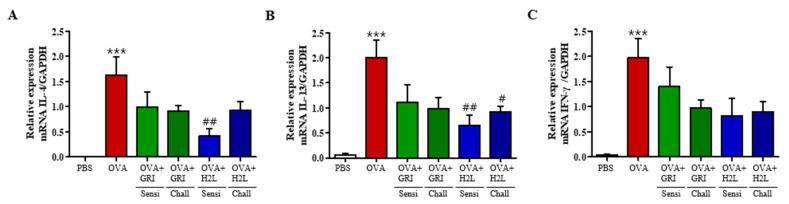
H2L5186303 and GRI977143 inhibit the mRNA expression of cytokines in the BALF cells. Analysis of mRNA expression of the Th2 cytokines IL-4 and IL-13 and the Th1 cytokine IFN-γ in BALF cells. (**A**) IL-4, (**B**) IL-13, and (**C**) IFN-γ levels. The relative mRNA levels of cytokines were quantified with respect to glyceraldehyde-3-phosphate dehydrogenase (GAPDH) as the housekeeping gene. Values are presented as the mean ± standard error (SE) (*n* = 5). *** *p* < 0.001 vs. the PBS-treated group, # *p* < 0.05, ## *p* < 0.01 vs. the OVA-treated group.

Th2 cells contribute to the pathogenesis of allergic asthma by producing Th2 cytokines, such as IL-4, IL-5, and IL-13, leading to eosinophil accumulation in the airway wall, mucus overproduction, and IgE synthesis. In addition, Th1 and Th17 cytokines are involved in late-stage pathogenesis of asthma. Therefore, changes in the mRNA levels of the Th2 cytokines IL-4 and IL-13, and Th1 cytokine IFN-γ were measured by qPCR in BALF cells. As shown in Figure 4, the mRNA levels of all three cytokines were increased in the BALF cells of the OVA group; however, H2L5186303 treatment before OVA sensitization or challenge significantly suppressed the elevated IL-4 and IL-13 levels (Figure 5). In addition, GRI977143 treatment before OVA sensitization or challenge suppressed the cytokine levels but to a non-significant level (Figure 5). This was consistent with the findings of mild suppression of BALF total cell count, lung inflammation, and mucin production after GRI977143 and H2L5186303 treatments before OVA sensitization or challenge.

**Figure 6:**
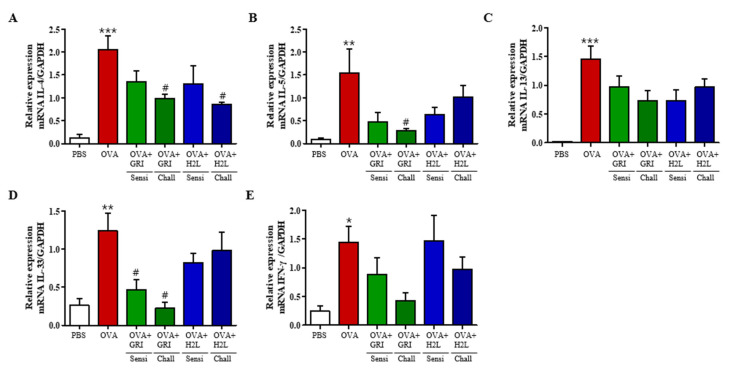
H2L5186303 and GRI977143 inhibit the mRNA expression of cytokines in the lungs. Analysis of mRNA expression of the Th2 cytokines IL-4, IL-5, IL-13, Th1 cytokine IL-33, and IFN-γ in lung tissues. (**A**) IL-4, (**B**) IL-5, (**C**) IL-13, (**D**) IL-33, and (**E**) IFN-γ. The relative mRNA levels of cytokines were quantified with respect to glyceraldehyde-3-phosphate dehydrogenase (GAPDH) as the housekeeping gene. Values are presented as the mean ± standard error (SE) (*n* = 5). * *p* < 0.05, ** *p* < 0.01, *** *p* < 0.001 vs. the PBS-treated group, # *p* < 0.05 vs. the OVA-treated group.

Furthermore, changes in the mRNA levels of Th2 and Th1 cytokines in the lungs were measured using qPCR. As shown in Figure 6, the mRNA levels of the five cytokines increased in the lungs of the OVA group, and GRI977143 treatment before OVA challenge significantly suppressed the elevated IL-4, IL-5, and IL-33 levels (Figure 6). However, H2L5186303 treatment before OVA sensitization did not significantly suppress the OVA-induced increase (Figure 6).

### 2.6. GRI977143 and H2L5186303 Treatments Suppressed OVA-Induced Elevated IL-13 Levels in BALF Unlike Serum IgE Levels

**Figure 7:**
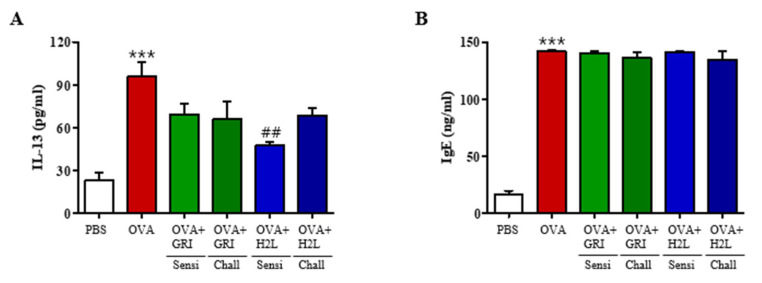
Effect of H2L5186303 and GRI977143 on IL-13 levels in the BALF and IgE levels in the serum. ELISA was used to measure the protein levels of (**A**) IL-13 in BALF and (**B**) IgE in serum. Results are presented as the mean ± standard error of the mean (SEM) (*n* = 5). *** *p* < 0.001 vs. the PBS-treated group, ## *p* < 0.01 vs. the OVA-treated group.

Th2 cytokines, such as IL-13 play a major role in the progression of allergic asthma. Th2 cytokines induce eosinophil recruitment and activation, mucus hypersecretion in epithelial cells, metaplasia of goblet cells, and the proliferation of smooth muscle cells. Protein levels of the Th2 cytokine IL-13 in the BALF were measured by ELISA, and results showed elevated IL-13 levels in the OVA-induced compared with those in the PBS-treated control group. Moreover, the elevated IL-13 levels were suppressed by GRI977143 and H2L5186303 treatments (Figure 7A). Furthermore, serum IgE levels were assessed to confirm the immunological effects induced by OVA, GRI977143, and H2L5186303 treatments. Results revealed that IgE levels were increased in the sera of OVA-treated mice (Figure 7B); however, these elevated levels were not suppressed by GRI977143 or H2L5186303 treatments before antigen sensitization and challenge.

## 3. Discussion

Previous findings have suggested various pro-allergic and pro-inflammatory responses of LPA as follows: (1) LPA inhalation induces histamine release and enhances eosinophil infiltration into alveolar spaces in guinea pigs [6,7]; (2) LPA not only induces chemotaxis of human eosinophils and chemokine generation through IL-4 in human mast cells but also enhances IL-13 gene expression in Th2 cells in vitro [8,10,11,12]; (3) LPA stimulated the expression of thymic stromal lymphopoietin and CCL20 in bronchial epithelial cells [15]; and (4) The LPA levels of C22:5 and C22:6 increased in the BALF of asthmatic patients and mice after allergen challenge [3,4,5]. Additionally, the pro-allergic roles of LPA were experimentally linked to LPA_2_ by three studies. Two studies showed a reduction in eosinophil influx and attenuation of Th2 cytokines in LPA_2_ gene-deficient mice (LPA_2_^+/−^ and LPA_2_^−/−^) [4,17], whereas a third study showed that H2L5186303 suppresses allergic airway inflammation [5]. Our results with the LPA_2_ antagonist H2L5186303 also support the pro-allergic responses of LPA and the role of LPA_2_. Kondo et al. showed that H2L5186303 improved AHR and reduced eosinophil infiltration and PAS-positive cell counts, suggesting that LPA_2_-mediated increase in CCL2 leads to macrophage activation and IL-33 production in allergic responses in the lung [5]. In the present study, we reproduced the previous findings of AHR improvement, eosinophil infiltration reduction, and suppression of mucin secretion. In addition, we reported two novel findings: (1) H2L5186303 suppressed mast cell degranulation, and (2) H2L5186303 was effective not only when delivered before antigen challenge but also before sensitization.

In contrast, several findings indicated LPA-mediated anti-allergic responses as follows: (1) LPA induced IL-13Rα2 (IL-13 decoy receptor) expression and release, and attenuated IL-13-induced phosphorylation of STAT6 in human bronchial epithelial cells [22]; (2) LPA attenuated cytokine secretion in human and murine dendritic cells [13,14]; (3) LPA_2_ gene-deficient mice (LPA_2_^−/−^) showed more lung inflammation than wild-type mice [19]; and (4) LPA_2_ agonist DBIBB suppressed allergic bronchial inflammation [21]. Emo et al. observed more severe allergen-driven lung inflammation in LPA_2_^−/−^ than in wild-type mice in allergic asthma models of both systemic and mucosal sensitization, in contrast to the above-mentioned experiments using LPA_2_^−/−^ mice [4,17,19]. Such discrepancies may result from difference in the origin of LPA_2_^−/−^ mice, i.e., Dr Chun’s lab vs. Deltagen, and their different adaptations to LPA_2_ gene deficiency. In addition, the experimental protocol used by Emo et al. was also different, where allergen-pulsed LPA_2_^−/−^ dendritic cells were adoptively transferred to wild-type mice owing to their hyperactivity in vitro [19]. This observation is consistent with the finding that LPA attenuates cytokine secretion in human and murine dendritic cells [13,14]. Consequently, LPA_2_ deficiency leads to cytokine hyper secretion due to a lack of LPA_2_ negative regulation. In this context, the results obtained with the LPA_2_ agonist DBIBB were consistent [21] in which HDM extract was delivered intranasally for 10 consecutive days and DBIBB was administered intraperitoneally [21]. Therefore, DBIBB was delivered during the antigen sensitization period, which suppressed the activation of dendritic cells. However, negative regulation via LPA_2_ was partly seen in our results with GRI977143. We observed significant AHR improvement, reduced eosinophils infiltration, and suppressed mucin secretion only with GRI977143 treatment before antigen challenge but not before sensitization, which is in contrast to previous observations of LPA_2_ negative regulation of dendritic cell activation. One intriguing point is that DBIBB can inhibit autotaxin activity, which produces LPA from lysophosphatidylcholine, implying that DBIBB not only activates LPA_2_ but also inhibits LPA production in the lung [21]. This may compound the results, as inhibition of LPA production could result in similar results as LPA_2_ antagonism. It seems that the LPA_2_ antagonist H2L5186303 did not inhibit autotaxin activity, as LPA levels in BALF were not affected by H2L5186303 [5].

Contradictory and contrasting results on LPA_2_ functions in allergic asthma models have been observed. We compared the results with the LPA_2_ agonist and antagonist in a single experimental protocol to gain a clear conclusion. Our findings revealed that the LPA_2_ antagonist H2L5186303 effectively suppresses the symptoms and immunologic responses in treatments before antigen sensitization and challenge. This supports the pro-allergic roles of LPA_2_ in allergic inflammation [4,5,17]. We also observed that the LPA_2_ agonist GRI977143 was effective in suppressing allergic responses only in treatment before antigen challenge, unlike the previously known negative regulation of dendritic cell activation by LPA_2_ [19,21]. The differences may result from the nature of allergens, such as HDM and OVA, the methods of sensitization and challenge, such as mucosal and systemic, the genetic backgrounds of the mice, such as C57BL/6 and BALB/c, and the timing of administration, such as before sensitization and challenge. In addition to the above-mentioned immune cells, bronchial epithelial cells and airway smooth muscle cells are also involved in allergic asthma reactions [16,23,24,25], which makes the interpretation of the results at the level of target cells and molecular mechanisms difficult. In addition, there are differences in LPA_2_ expression between mice and humans [16].

Conclusively, the present results demonstrate multiple roles of LPA_2_ in asthmatic responses and suggest that the development of LPA_2_ antagonists would achieve better therapeutic efficacy against asthma than agonists, although it would be a challenge to develop a therapeutic targeting LPA_2_. However, local delivery might be an option to reduce systemic side effects.

## 4. Materials and Methods

### 4.1. Materials

H2L5186303, GRI977143, OVA, and alum were procured from Sigma-Aldrich (St. Louis, MO, USA).

### 4.2. Cell Culture

Rat RBL-2H3 mast cells were obtained from the American Type Culture Collection (ATCC, Manassas, VA, USA) and cultured in Dulbecco’s modified Eagle medium (DMEM)-high glucose containing 10% (*v*/*v*) heat-inactivated fetal bovine serum along with 2 mM glutamine, 100 U/mL penicillin, 1 mM sodium pyruvate, and 50 μg/mL streptomycin at 37 °C in a 5% CO_2_-humidified incubator.

### 4.3. Animals

Five-week-old female BALB/c mice were purchased from Daehan Biolink (Seoul, Korea) and maintained in the laboratory animal facility at Kyung Hee University by providing water and food *ad libitum*. The Institutional Animal Care Committee of the university reviewed and approved the study protocol, considering the ethical principles and the care and use of animals for scientific purposes (Approval Number, KHSASP-21-352).

### 4.4. Assessment of Degranulation

Degranulation of RBL-2H3 cells was assessed by measuring β-hexosaminidase activity in the medium. Mouse monoclonal anti-dinitrophenyl immunoglobulin E (DNP-IgE) and human DNP albumin were used to induce degranulation [26].

### 4.5. Asthma Induction in Mice and Administration of H2L5186303 and GRI977143

Following a simple randomization procedure, six-week-old female BALB/c mice (approximately 22 g) were randomly assigned to six treatment groups (*n* = 5): PBS-injected control group, OVA-injected asthma group, GRI977143 (1 mg/kg) treatment before sensitization plus OVA-injected group, GRI977143 treatment before challenge plus OVA-injected group, H2L5186303 (1 mg/kg) treatment before sensitization plus OVA-injected group, and H2L5186303 treatment before challenge plus OVA-injected group. Asthma was induced by intraperitoneal injection of 50 μg OVA and 1 mg aluminum hydroxide on days 0 (D0) and 14 (D14; sensitization). The mice were challenged with nebulized OVA on D28, D29, and D30 (challenge). GRI977143 or H2L5186303 was administered via intraperitoneal injection 30 min before OVA sensitization or challenge. BALF was collected from the lung tissues on D32 followed by staining for cell population analysis [27].

### 4.6. BALF Cell Counting and Analysis

Immune cells in BALF were adhered to a glass slide using a Cellspin^®^ centrifuge (Hanil Electric, Seoul, Republic of Korea) and fixed in methanol for 30 s. Cells were stained with May–Grünwald solution for 8 min, followed by Giemsa solution for 12 min.

### 4.7. Histological Examination of the Lung Tissues

Lung sections from mice in each group were prepared and examined. Briefly, left lungs were fixed in 10% formalin and dehydrate 30% sucrose solution and embedded in O.C.T. compound. Sections (7 µm) were stained with hematoxylin and eosin (H&E), and periodic acid-Schiff (PAS; using Schiff’s reagent) stain to identify eosinophil infiltration and mucus-secreting cells (goblet cells) in the airways, respectively. The right lung tissue was crushed, put in 1 mL of trizol, stored, extracted and synthesized and then confirmed by qPCR.

The degree of lung inflammation was estimated by a treatment-blinded observer using a subjective scale of 0–3. PAS-stained mucin-secreting cells in the airways were counted in two lung sections per mouse. Consequently, mucous production was expressed as the number of PAS-positive cells per millimeter of bronchiole. Moreover, the length of the bronchial basal lamina was measured using ImageJ software (National Institute of Health).

### 4.8. Measurement of Airway Hyperresponsiveness

Airway hyper-responsiveness (AHR) was detected using noninvasive whole-body plethysmography (Model PLY-UNR-MS2; EMKA Technologies, Paris, France) 24 h after the last OVA treatment.

### 4.9. Measurement of Total Serum IgE and IL-13 Level

Mouse serum IgE and IL-13 levels were determined using ELISA kits (eBioscience, San Diego, CA, USA). IL-13 specific capture and biotinylated antibodies were obtained from eBioscience (IL-4: Cat. No. 14-7041-68 and 33-7042-68C, IL-13: Cat no. 14-7043-68 and 33-7135-68B; San Diego, CA, USA). Avidin-horseradish peroxidase was used, and the absorbance was measured at 450 nm.

### 4.10. Statistical Analysis

Statistical analysis was performed using GraphPad Prism software (GraphPad Software, Inc., La Jolla, CA, USA). Data were expressed as mean ± standard error of the mean (SEM). One-way analysis of variance (ANOVA) followed by Tukey’s multiple comparison test was used to compare the differences among multiple groups. Differences were considered statistically significant at *p* < 0.05.
